# Predictive and prognostic factors of septic shock of nosocomial origin

**DOI:** 10.1186/cc10656

**Published:** 2012-03-20

**Authors:** JP Quenot, A Pavon, C Binquet, F Kara, O Martinet, F Ganster, JC Navellou, V Castelain, D Barraud, J Cousson, JF Poussel, P Perez, K Kuteifan, A Noirot

**Affiliations:** 1 University Hospital Bocage, Dijon, France; 2 Centre Hospitalier, Haguenau, France; 3 Nouvel Hopital Civil, Strasbourg, France; 4 University Hospital, Besancon, France; 5 Hopital Hautepierre, Strasbourg, France; 6 Hopital Central, Nancy, France; 7 Regional Hospital, Metz-Thionville, France; 8 Hopital Brabois, Nancy, France; 9 CHG, Mulhouse, France

## Introduction

The incidence of septic shock in intensive care in France is around 8 to 10%, with in-hospital mortality ranging from 55 to 60% [1]. Mortality increases by 10% when the infection causing septic shock is acquired in-hospital or in the ICU [1]. We aimed to determine predictive and prognostic factors for septic shock caused by a nosocomial infection (NI).

## Methods

Subgroup analysis of a prospective, multicentre, observational study performed between November 2009 and March 2011 in 14 ICUs from 10 university and community (nonacademic) hospitals in the northeast of France. This study was supported by the Collège Interrégional des Réanimateurs du Nord-Est. Patients were included if they were aged >18 years and had septic shock plus at least one criterion of hypoperfusion. Infection was classed as nosocomial if acquired in-hospital more than 48 hours after admission. Data control and statistical analysis were performed by the CIC-EC of Dijon University Hospital (INSERM Unit CIE1).

## Results

In total, 1,147 patients were included in the cohort, of whom 409 (35.6%) presented a NI (345/409 (84%) acquired in-hospital and 64/409 (16%) acquired in the ICU). The factors significantly associated with NI (in-hospital or in-ICU) were: immunodepression, a Knaus score C to D, SAPS II score, and SOFA score. Other variables such as age, sex, type of admission and type of infection were not significantly related to the nosocomial origin of infection. In-hospital mortality for community-acquired versus NIs was 40.8% vs. 53.5% respectively (*P *< 0.01), and 46.9% vs. 62% respectively at 28 days (*P *< 0.01).

## Conclusion

Mortality of patients with septic shock of nosocomial origin is particularly high. Scores evaluating gravity of disease are also higher in patients with NI versus those with community-acquired infection. This could be explained by delayed presentation or difficulties with management, but also by immunodepression and a poor state of prior health. It is likely that appropriate measures, particularly aimed at prevention, could help to reduce mortality in patients with septic shock caused by NI.
